# Fetal growth-guided management of gestational diabetes: A retrospective cohort study on insulin requirements and perinatal outcomes

**DOI:** 10.1007/s00404-026-08467-3

**Published:** 2026-05-21

**Authors:** Serena Xodo, Andrea Da Porto, Maria De Martino, Annalaura Del Pin, Asia Flaiban, Silvia Galasso, Sara Piccini, Lorenza Driul

**Affiliations:** 1 Clinic of Obstetrics and Gynecology, Santa Maria Della Misericordia Hospital, Azienda Sanitaria Universitaria Friuli Centrale (ASUFC), Udine, Italy; 2 Department of Internal Medicine, Diabetes and Metabolism Unit, Health Integrated Agency of Friuli Centrale, 33100 Udine, Italy; 3 Department of Medical Area, University of Udine, Udine, Italy; 4 Division of Medical Statistic, Department of Medicine (DMED), University of Udine, Udine, Italy; 5 Department of Neurobiology, Care Sciences and Society, Aging Research Center, Karolinska Institutet and Stockholm University, Stockholm, Sweden

**Keywords:** Gestational diabetes, Ultrasonographic fetal growth, Fetal abdominal circumference, Insulin therapy, Personalized therapy

## Abstract

**Purpose:**

To evaluate whether a fetal growth-guided management strategy can reduce treatment intensity without compromising maternal and neonatal outcomes in women with gestational diabetes mellitus (GDM).

**Methods:**

This retrospective cohort study with propensity score matching was conducted at a single tertiary referral center and included 343 women with GDM diagnosed after a second-trimester OGTT, of whom 244 were included in the matched analysis. Participants were managed either with a fetal growth-guided approach, in which glycemic targets and insulin therapy were tailored according to fetal abdominal circumference (AC), or with standard glycemia-based care. In the growth-guided group, stricter targets were applied when AC exceeded the 70th percentile. Outcomes were analyzed in the overall cohort and after matching. Primary outcomes were insulin use and glycemic control. Secondary outcomes included maternal weight gain, obstetric outcomes, and neonatal outcomes such as birthweight, Apgar score, cord blood pH, and size for gestational age.

**Results:**

In the overall cohort, fetal growth-guided management was associated with lower birthweight (3470 g vs 3544 g; p = 0.036), reduced basal insulin use (58% vs 83%; p < 0.001), lower total insulin dose at delivery (9 vs 18 IU; p < 0.001), and slightly improved glycemic control. After propensity score matching, basal insulin use (65% vs 80%; p = 0.029) remained lower, while glycemic control, obstetric outcomes, and neonatal outcomes were comparable. The sensitivity analysis restricted to insulin-treated patients ahowed no significant differences between the differently managed groups of patients.

**Conclusions:**

A fetal growth-guided approach to GDM management reduces insulin requirements without worsening maternal or neonatal outcomes.

## What does this study adds to the clinical work

**Table:** 

This study suggests that integrating fetal growth assessment into gestational diabetes management may reduce insulin requirements while maintaining comparable maternal and neonatal outcomes. A fetal growth-guided approach could support more personalized treatment and help avoid unnecessary treatment intensification.	

## Introduction

Gestational diabetes mellitus (GDM) is one of the most common medical complications of pregnancy and represents a major contributor to adverse perinatal outcomes [1]. Maternal hyperglycemia is strongly associated with excessive fetal growth, macrosomia, shoulder dystocia, and neonatal metabolic complications [1, 2]. Consequently, contemporary management strategies focus on achieving strict glycemic targets through dietary intervention and, when required, pharmacological therapy [3]. This approach assumes a relatively homogeneous fetal response to maternal hyperglycemia, although clinical and biological evidence suggests substantial variability in fetal susceptibility to the diabetic intrauterine environment [4]. As a result, some pregnancies may be exposed to unnecessarily intensive treatment, whereas others may remain at risk of excessive fetal growth despite apparently adequate glycemic control.

Fetal growth assessment, particularly fetal abdominal circumference (AC), has been proposed as a clinically meaningful marker of fetal metabolic exposure, reflecting the effects of fetal hyperinsulinaemia and altered nutrient transfer [5]. On this basis, a strategy of fetal growth guided management has been suggested, in which the intensity of metabolic control is tailored according to fetal biometry. In pregnancies with evidence of accelerated fetal growth, tighter glycemic targets and earlier treatment intensification may be adopted, while a more conservative therapeutic approach may be considered when fetal growth remains within normal limits. Despite its biological rationale, the clinical utility of this strategy remains uncertain. A Cochrane review found limited and inconclusive evidence regarding the effectiveness of fetal biometry-guided management in improving maternal and perinatal outcomes in GDM [6]. In this context, further evidence from real-world clinical settings is needed to clarify whether integrating fetal growth parameters into the therapeutic decision-making process can meaningfully influence treatment intensity, obstetric management, and perinatal outcomes. The aim of the present study was therefore to evaluate the impact of a fetal growth-guided management strategy compared with standard glycemia-based care in women with GDM.

## Methods

We conducted a retrospective observational study including women diagnosed with GDM, who underwent a labor induction (IOL) at a single tertiary obstetric referral center between January 2023 and December 2023. GDM was diagnosed using a 75-g oral glucose tolerance test (OGTT). Early screening at 16–18 weeks of gestation was offered to women presenting at least one of the following risk factors: (i) GDM in a previous pregnancy; (ii) body mass index (BMI) ≥ 30 kg/m^2^; or (iii) pre-pregnancy or early pregnancy fasting plasma glucose (FPG) between 100 and 125 mg/dL. If the initial screening was negative, the OGTT was repeated at 24–28 weeks of gestation. According to the local protocol, women were also offered OGTT at 24–28 weeks if they presented at least one of the following risk factors: (i) maternal age ≥ 35 years; (ii) pre-pregnancy BMI ≥ 25 kg/m^2^; (iii) previous fetal macrosomia (birth weight ≥ 4.5 kg); (iv) GDM in a previous pregnancy; (v) family history of type 2 diabetes in a first-degree relative; or (vi) origin from South Asia or the Middle East. The diagnostic cut-off values for the OGTT were those derived from the IADPSG/WHO criteria, as adopted in the Italian national guidelines for the diagnosis of GDM [7, 8]. After diagnosis, patients were managed by a multidisciplinary team including obstetricians and diabetologists. Obstetric care included monthly fetal ultrasound assessments from the time of diagnosis, whereas diabetological follow-up consisted of monthly clinical evaluations, with visits every two weeks in cases of inadequate glycemic control. All ultrasound examinations were performed by experienced obstetric sonographers using Voluson E8 and Voluson E10 ultrasound systems. Fetal biometric measurements were interpreted according to the Hadlock reference growth curves [9].

The study population was divided into two groups according to the type of diabetes management: personalized management, in which fetal growth parameters were incorporated into therapeutic decision-making, and standard management, in which fetal growth was not considered in treatment adjustment. In the personalized management group, glycemic targets and the intensity of insulin therapy were adjusted according to fetal AC. Specifically, when AC exceeded the 70th percentile, glycemic targets were tightened (90 mg/dL fasting and 130 mg/dL after meals), consequently insulin therapy was intensified if necessary; conversely, when AC was equal or below this threshold, less stringent glycemic targets** (**95 mg/dl fasting and 140 mg/dl after meals) were adopted and insulin therapy was adjusted less aggressively. All diabetological consultations during pregnancy were reviewed for each patient. Patients were assigned to the personalized management group if fetal growth parameters (AC and estimated fetal weight, EFW) were documented at every consultation. Those in whom fetal biometric parameters were not recorded in at least two consultations were classified in the standard management group. Only singleton pregnancies with a confirmed diagnosis of GDM and available obstetric and neonatal outcome data were included in the analysis. Women with pregestational diabetes were excluded.

Obstetric and neonatal outcomes were retrieved from electronic medical records. Participants were categorized according to the Robson Ten-Group Classification System [10]. Obstetric outcomes included gestational age at delivery, IOL characteristics, and mode of delivery, categorized as spontaneous vaginal delivery (SVD), operative vaginal delivery (OVD), or cesarean delivery (CD). Additional obstetric variables comprised the duration of the different phases of labor and induction-related parameters, including the methods used at each step of the IOL process. Neonatal outcomes included birth weight, birth weight percentile according to specific reference charts [11], Apgar score at 1 and 5 min, umbilical artery gas values (pH and BE), and admission to the neonatal intensive care unit (NICU). The rate of neonates classified as large of gestational age (LGA) and small for gestational age (SGA) was reported for each group. The study protocol was approved by the local Institutional Review Board (DAME-IRB 333/2024) on November 8th, 2024. The study was conducted in accordance with the principles of the Declaration of Helsinki [12]. Due to the retrospective design and the use of anonymized data, the requirement for informed consent was waived.

## Statistical analysis

Continuous variables are reported as median and interquartile range (IQR) or mean ± standard deviation (SD), as appropriate. Categorical variables are presented as absolute counts and percentages. Group comparisons were performed using the Wilcoxon rank-sum test or Student’s t-test for continuous variables and the chi-square or Fisher’s exact test for categorical variables, as appropriate. To reduce confounding, a propensity score matching (PSM) analysis was performed. The propensity score was estimated using a multivariable logistic regression model with the treatment group (fetal growth-guided management vs standard management) as the dependent variable and maternal weight at the first diabetology consultation, prior GDM, fasting OGTT glucose (OGTT-T0) and Robson classification as the independent variables, selected a priori based on their clinical relevance. Patients were matched 1:1 using nearest-neighbor matching without replacement. Covariate balance after matching was assessed by comparing baseline characteristics between groups. The matched cohort was then used for subsequent analyses. All tests were two-sided, and a p-value < 0.05 was considered statistically significant. All analyses were conducted using R (version 4.5.1). Propensity score matching was performed using the MatchIt package (version 4.7.2).

## Results

A total of 343 women diagnosed with GDM and scheduled for IOL were included in the final analysis: 206 in the personalized management group and 137 in the standard management group. Maternal age at delivery was similar between the two groups. Table 1 summarizes baseline maternal characteristics, glycemic parameters, insulin therapy, obstetric management, and neonatal outcomes in the overall cohort. Regarding baseline maternal characteristics, pre-pregnancy weight was significantly higher in the standard management group (78 [65–91] kg vs 68 [60–79] kg, p < 0.001), as were weight at the first diabetologic consultation (84 [72–95] kg vs 77 [68–88] kg, p = 0.002) and weight at delivery (87 [76–99] kg vs 80 [72–91] kg, p < 0.001). No significant differences were observed in gestational weight gain. Previous GDM was more frequent among women in the standard management group (35% vs 18%, p = 0.001), whereas pregnancies achieved through assisted reproductive technologies (ART) were more common in the personalized management group (19% vs 6.7%, p = 0.035).

**Table 1:** Baseline maternal characteristics, glycemic parameters, insulin therapy, obstetric management, and neonatal outcomes in the overall cohort, comparing fetal growth–guided management versus standard glycemia-based management in women with gestational diabetes.

Characteristic	Fetal growth guided -management N = 206^*1*^	Standard Management N = 137^*1*^	p-value^*2*^
ART pregnancy	17/90 (19%)	4/60 (6.7%)	0.035
Maternal age at delivery (years)	34.0 (30.0, 38.0)	34.0 (30.0, 38.0)	0.390
Unknown	0	2	
Gestational age at delivery time (weeks)	39.00 (39.00, 40.00)	39.00 (39.00, 39.00)	0.005
Unknown	0	2	
Gestational age at delivery time (total days)	277.0 (273.0, 280.0)	276.0 (273.0, 279.0)	0.008
Pre-gestational weight (kg)	68 (60, 79)	78 (65, 91)	< 0.001
Unknown	71	48	
Maternal weight at first diabetologic consultation (kg)	77 (68, 88)	84 (72, 95)	0.002
Unknown	21	15	
Maternal weight at delivery time (kg)	80 (72, 91)	87 (76, 99)	< 0.001
Unknown	24	16	
Previous GDM	33/183 (18%)	42/119 (35%)	0.001
Fasting blood glucose in first trimester (mg/dL)	84 (79, 98)	106 (96, 122)	0.052
Unknown	200	129	
OGTT_T0 (mg/dL)	94 (91, 98)	97 (93, 102)	< 0.001
Unknown	33	23	
OGTT_T60 (mg/dL)	161 (134, 185)	157 (139, 188)	0.908
Unknown	34	26	
OGTT_T120 (mg/dL)	129 (110, 155)	127 (111, 146)	0.636
Unknown	34	26	
HbA1c (%)	5.20 (4.90, 5.50)	5.40 (5.10, 5.60)	0.086
Basal Insulin (IU)	106/184 (58%)	101/121 (83%)	< 0.001
Prandial Insulin (IU)	28/184 (15%)	28/121 (23%)	0.080
Total Insulin dosage at delivery (IU)	9 (3, 18)	18 (8, 26)	< 0.001
Unknown	56	23	
90-day average self-monitored blood glucose (mg/dL)	106 (102, 111)	108 (104, 113)	0.028
Unknown	31	17	
90-day average self-monitored blood glucose SD	19.1 (16.6, 22.4)	19.7 (17.2, 23.2)	0.261
Unknown	32	19	
Dietitian management	47/183 (26%)	48/121 (40%)	0.010
Urinary ketones	30/180 (17%)	19/118 (16%)	0.898
Gestational age at anatomical scan (weeks)	20.00 (20.00, 20.00)	20.00 (20.00, 20.00)	0.405
Unknown	33	16	
AC_anatomical scan (mm)	158 (152, 165)	159 (153, 164)	0.548
Unknown	33	16	
AC_percentile (Hadlock) (%)	74 (44, 91)	76 (43, 92)	0.711
Unknown	34	16	
Gestational age last scan in third trimester (weeks)	36.00 (35.00, 37.00)	36.00 (35.00, 37.00)	0.936
Unknown	34	14	
EFW (grams)	2903 (2553, 3213)	3023 (2705, 3314)	0.051
Unknown	48	22	
EFW_percentile (Hadlock) (%)	60 (46, 84)	67 (41, 87)	0.263
Unknown	65	42	
AC_last scan (mm)	324 (308, 340)	327 (311, 343)	0.370
Unknown	58	34	
AC_last scan percentile (Hadlock) (%)	74 (53, 91)	74 (54, 92)	0.658
Unknown	56	32	
Number diabetologic consultations (n)	5.00 (4.00, 6.00)	6.00 (5.00, 8.00)	< 0.001
Number fetal growth scans in pregnancy (n)	4.00 (3.00, 6.00)	3.00 (2.00, 5.00)	< 0.001
At least one consultation without considering fetal growth pattern (n)	55 (27%)	137 (100%)	< 0.001
IOL interruption	20/66 (30%)	12/45 (27%)	0.678
IOL duration (min)	1366 (645, 2566)	1556 (740, 2798)	0.774
Unknown	15	10	
Active first stage of labor duration (min)	135 (71, 275)	125 (55, 240)	0.288
Unknown	35	23	
Second stage of labor duration (min)	30 (13, 70)	24 (11, 60)	0.293
Unknown	42	27	
Induction – labor onset duration (min)	970 (450, 1,980)	1040 (585, 2400)	0.221
Unknown	27	20	
Spontaneous vaginal delivery	150 (73%)	106/135 (79%)	0.234
Operative vaginal delivery	19 (9.2%)	6/135 (4.4%)	0.098
Cesarean delivery	37 (18%)	23/135 (17%)	0.827
Robson			0.006
2 (Nulliparous women with a single cephalic pregnancy, ≥ 37 weeks, induced labor or pre-labor cesarean section)	107 (52%)	50/135 (37%)	
3 (Multiparous women without previous cesarean, single cephalic pregnancy, ≥ 37 weeks, spontaneous labor)	1 (0.5%)	0/135 (0%)	
4 (Multiparous women without previous cesarean, single cephalic pregnancy, ≥ 37 weeks, induced labor or pre-labor cesarean section)	83 (40%)	76/135 (56%)	
5 (All multiparous women with at least one previous cesarean section, single cephalic pregnancy, ≥ 37 weeks)	15 (7.3%)	7/135 (5.2%)	
9 (All women with a single pregnancy with transverse or oblique lie (including previous cesarean)	0 (0%)	2/135 (1.5%)	
Apgar 5 min	9.00 (9.00, 9.00)	9.00 (9.00, 10.00)	0.618
Unknown	1	4	
Umbilical vein pH at birth	7.28 (7.24, 7.33)	7.27 (7.23, 7.32)	0.439
Unknown	18	15	
Umbilical vein BE at birth	−2.9 (−5.1, −1.3)	−3.5 (−5.5, −1.5)	0.473
Unknown	98	70	
Neonatal weight at birth (grams)	3470 (3190, 3730)	3544 (3295, 3765)	0.036
Unknown	1	2	
Neonatal weight percentile (Bertino) (%)	58 (31, 82)	66 (45, 87)	0.020
Unknown	1	0	
LGA neonates	35 (17%)	27 (20%)	0.535
Unknown	1	0	
SGA neonates	13 (6.3%)	6 (4.4%)	0.438
Unknown	1	0	
NICU admission	4 (1.9%)	4 (2.9%)	0.718
Post-partum hemorrhage	35/205 (17%)	23/135 (17%)	0.993

^*1*^ Median (Q1, Q3); n (%)

^*2*^ Wilcoxon rank sum test; NA; Fisher’s exact test; Pearson’s Chi-squared test; Wilcoxon rank sum exact test

*ART* assisted reproductive technology, *GDM* gestational diabetes mellitus, *OGTT* Oral glucose tolerance test, *SD*  standard deviation, *AC*  abdominal circumference, *EFW*   estimated fetal weight, *IOL*   induction of labor, *LGA* Large for gestational age, *SGA*   small for gestational age, *NICU*  neonatal intensive care unit

Regarding OGTT results, fasting glucose values were significantly higher in the standard management group (97 [93–102] mg/dL vs 94 [91–98] mg/dL, p < 0.001). In contrast, glucose levels at 60 min and 120 min and HbA1c values were comparable between the groups. With regard to insulin therapy, basal insulin was more frequently prescribed in the standard management group (83% vs 58%, p < 0.001), while prandial insulin showed a non-significant trend toward higher use. The total insulin dose at delivery was significantly higher in the standard management group (18 [8–26] IU vs 9 [3–18] IU, p < 0.001). Mean self-monitored glucose levels during the last 90 days of pregnancy were slightly higher in the standard management group (108 [104–113] mg/dL vs 106 [102–111] mg/dL, p = 0.028), whereas glycemic variability did not differ between groups.

As for delivery characteristics, gestational age at delivery was slightly but significantly lower in the standard management group (276 [273–279] days vs 277 [273–280] days, p = 0.008). The distribution of Robson classification groups differed between the cohorts (p = 0.006), with a higher proportion of group 4 in the standard management group (multiparous women without a previous cesarean, with a singleton, term, cephalic pregnancy, who have labor induced or undergo cesarean before labor) and group 2 in the personalized management group (nulliparous women with a singleton, term, cephalic pregnancy, who have labor induced or undergo cesarean before labor). However, the duration of induction, the time from induction to active labor, the duration of the expulsive phase and the rate of IOL interruption were similar between groups. Finally, delivery mode and neonatal outcomes were largely comparable between groups. The rates of SVD, OVD, and CD were 73% vs 79%, 9.2% vs 4.4%, and 18% vs 17%, respectively. Neonatal outcomes, including Apgar scores at 1 and 5 min, umbilical cord pH, and base excess, were similar between groups. However, birthweight was slightly higher in the standard management group (3544 [3295–3765] g vs 3470 [3190–3730] g, p = 0.036), as was the birthweight percentile according to Bertino charts (66 [45–87] vs 58 [31–82], p = 0.020). The rate of neonatal transfer to NICU and the incidence of postpartum hemorrhage were comparable between groups, with no significant differences. The rates of LGA and SGA neonates were similar between groups.

After multivariable propensity score matching, 186 women were included in the analysis: 93 pregnancies in which GDM management depended on fetal growth and 93 in which it did not. Table 2 illustrates baseline characteristics, metabolic control, treatment intensity, obstetric outcomes, and neonatal outcomes in the propensity score-matched cohort. Baseline characteristics were well balanced between groups after matching. In particular, key confounders, such as previous GDM, fasting OGTT glucose (OGTT-T0), and Robson classification, showed no significant differences between groups, indicating adequate covariate balance. Maternal age at delivery, gestational age, and anthropometric characteristics were also comparable. As for GDM management, basal insulin therapy was more frequently used in the standard management group (80% vs 65%, p < 0.029), while prandial insulin and the total insulin dose at delivery was similar between groups. The fetal growth-guided group underwent a higher number of obstetric ultrasound examinations, whereas diabetologic consultations were more frequent in the standard management group (both p < 0.001). Glycemic control, as assessed by mean glucose values and glycemic variability, did not differ significantly between groups. Obstetric outcomes were comparable between groups. Gestational age at delivery, duration of induction, active labor, and the expulsive phase were comparable between groups. The mode of delivery did not significantly differ: SVD occurred in 76% vs 78% of cases, OVD in 4.3% vs 3.2%, and CD in 19% vs 18%. Similarly, neonatal outcomes were comparable, including Apgar score at 1 and 5 min, umbilical artery pH, neonatal weight (3495 [3180–3760] vs 3525 [3295–3745] g, p = 0.367), birthweight percentile, and NICU admission. After matching, LGA rates remained comparable, while SGA showed a non-significant trend toward higher prevalence in the fetal growth-guided group (5.4% vs 3.2%, p = 0.721).

**Table 2:** Baseline characteristics, metabolic control, treatment intensity, obstetric outcomes, and neonatal outcomes in the propensity score–matched cohort, comparing fetal growth–guided management versus standard management.

Characteristic		Fetal growth guided -management N = 93^*1*^	Standard management N = 93^*1*^	p-value^*2*^
ART pregnancy		6 (15%)	4 (8.5%)	0.504
Maternal age at delivery (years)		34.0 (30.0, 37.0)	35.0 (30.0, 38.0)	0.139
Gestational age at delivery (weeks)		39.00 (39.00, 39.00)	39.00 (39.00, 39.00)	0.685
Gestational age at delivery (days)		277.0 (274.0, 279.0)	276.0 (273.0, 279.0)	0.465
Pregestational weight (kg)		74 (64, 84)	75 (64, 89)	0.803
Maternal weight at first diabetologic consultation (kg)		81 (72, 94)	82 (70, 91)	0.805
Maternal weight at delivery (kg)		84 (75, 95)	85 (74, 98)	0.796
Delta weight		11.1 (7.4, 14.2)	10.6 (6.5, 13.4)	0.730
Previous GDM		20 (22%)	28 (31%)	0.152
OGTT_T0		95 (93, 99)	95 (93, 99)	0.771
OGTT_T60		157 (136, 180)	157 (139, 186)	0.744
Unknown		0	2	
OGTT_T120		122 (109, 147)	125 (110, 146)	0.665
Unknown		0	2	
HbA1c		5.20 (4.90, 5.50)	5.20 (4.90, 5.40)	0.606
Unknown		62	51	
Basal Insulin		60 (65%)	74 (80%)	0.029
Unknown		1	0	
Prandial Insulin		16 (17%)	14 (15%)	0.666
Unknown		1	0	
Total Insulin dosage at delivery		9 (4, 19)	12 (6, 24)	0.090
Unknown		11	6	
90 day average self-monitored blood glucose		106 (102, 113)	108 (104, 112)	0.263
Unknown		4	0	
90 day average self-monitored blood glucose SD		18.7 (16.1, 22.3)	19.2 (17.2, 22.3)	0.475
Unknown		4	2	
Dietitian management		27 (29%)	29 (31%)	0.786
Unknown		1	0	
Urinary ketones		16 (18%)	14 (16%)	0.714
Unknown		2	3	
Gestational age at anatomical scan (weeks)		20.00 (20.00, 20.00)	20.00 (20.00, 20.00)	0.728
Unknown		15	14	
AC_anatomical scan		160 (153, 165)	160 (155, 164)	0.950
Unknown		15	14	
AC_percentile (Hadlock)		77 (47, 90)	76 (49, 93)	0.877
Unknown		16	14	
Gestational age at last scan in third trimester (weeks)		36.00 (35.00, 37.00)	36.00 (35.00, 37.00)	0.333
Unknown		15	11	
EFW (grams)		2915 (2651, 3223)	3014 (2681, 3252)	0.461
Unknown		21	16	
EFW percentile (Hadlock)		73 (47, 85)	64 (38, 82)	0.398
Unknown		35	31	
AC_last scan		326 (311, 340)	324 (311, 341)	0.725
Unknown		30	26	
AC_last scan percentile (Hadlock)		80 (54, 93)	70 (52, 90)	0.385
Unknown		31	27	
Number of diabetologic consultations		5.00 (4.00, 6.00)	6.00 (5.00, 7.00)	0.000
Number of fetal growth scans in pregnancy		5.00 (4.00, 6.00)	3.00 (2.00, 4.00)	0.000
At least one consultation without considering fetal growth pattern		29 (31%)	93 (100%)	0.000
DELTA_AC_HADLOCK		2 (−5, 11)	2 (−14, 22)	0.963
Unknown		36	38	
IOL interruption	9 (28%)		8 (26%)	0.836
IOL duration (min)	1685 (707, 2967)		1498 (703, 2407)	0.143
Unknown	6		7	
Active first stage of labor duration (min)	125 (71, 235)		125 (54, 180)	0.535
Unknown	16		15	
Second stage of labor duration (min)	22 (11, 51)		28 (11, 61)	0.735
Unknown	19		18	
Induction – labor onset duration (min)	1260 (460, 2311)		996 (442, 2013)	0.362
Unknown	12		13	
Spontaneous vaginal delivery	71 (76%)		73 (78%)	0.726
Operative vaginal delivery	4 (4.3%)		3 (3.2%)	1.000
Cesarean delivery	18 (19%)		17 (18%)	0.851
Robson				0.711
2 (Nulliparous women with a single cephalic pregnancy, ≥ 37 weeks, induced labor or pre-labor cesarean section)	40 (43%)		37 (40%)	
4 (Multiparous women without previous cesarean, single cephalic pregnancy, ≥ 37 weeks, induced labor or pre-labor cesarean section)	46 (49%)		51 (55%)	
5 (All multiparous women with at least one previous cesarean section, single cephalic pregnancy, ≥ 37 weeks)	7 (7.5%)		5 (5.4%)	
Apgar 1 min	8.00 (8.00, 9.00)		8.00 (8.00, 9.00)	0.687
Apgar 5 min				0.660
4	1 (1.1%)		0 (0%)	
6	1 (1.1%)		0 (0%)	
7	1 (1.1%)		2 (2.2%)	
8	8 (8.7%)		11 (12%)	
9	59 (64%)		53 (58%)	
10	21 (23%)		26 (28%)	
Umbilical artery pH at birth	7.28 (7.24, 7.32)		7.28 (7.24, 7.32)	0.788
Unknown	11		9	
Umbilical artery BE at birth	−2.75 (−5.10, −1.30)		−3.60 (−5.30, −1.80)	0.342
Unknown	43		47	
Neonatal weight at birth (grams)	3495 (3180, 3760)		3525 (3295, 3745)	0.367
Neonatal weight percentile (Bertino)	60 (28, 86)		64 (43, 86)	0.192
LGA neonates	20 (22%)		14 (15%)	0.255
SGA neonates	5 (5.4%)		3 (3.2%)	0.721
NICU admission	2 (3.5%)		1 (1.7%)	0.615
Post-partum hemorrhage	18 (20%)		17 (18%)	0.823

Characteristic		Fetal growth guided -management N = 93^*1*^	Standard management N = 93^*1*^	p-value^*2*^
ART pregnancy		6 (15%)	4 (8.5%)	0.504
Maternal age at delivery (years)		34.0 (30.0, 37.0)	35.0 (30.0, 38.0)	0.139
Gestational age at delivery (weeks)		39.00 (39.00, 39.00)	39.00 (39.00, 39.00)	0.685
Gestational age at delivery (days)		277.0 (274.0, 279.0)	276.0 (273.0, 279.0)	0.465
Pregestational weight (kg)		74 (64, 84)	75 (64, 89)	0.803
Maternal weight at first diabetologic consultation (kg)		81 (72, 94)	82 (70, 91)	0.805
Maternal weight at delivery (kg)		84 (75, 95)	85 (74, 98)	0.796
Delta weight (kg)		11.1 (7.4, 14.2)	10.6 (6.5, 13.4)	0.730
Previous GDM		20 (22%)	28 (31%)	0.152
OGTT_T0 (mg/dL)		95 (93, 99)	95 (93, 99)	0.771
OGTT_T60 (mg/dL)		157 (136, 180)	157 (139, 186)	0.744
OGTT_T120 (mg/dL)		122 (109, 147)	125 (110, 146)	0.665
HbA1c (%)		5.20 (4.90, 5.50)	5.20 (4.90, 5.40)	0.606
Unknown		62	51	
Basal Insulin (IU)		60 (65%)	74 (80%)	0.029
Prandial Insulin (IU)		16 (17%)	14 (15%)	0.666
Total Insulin dosage at delivery (IU)		9 (4, 19)	12 (6, 24)	0.090
Unknown		11	6	
90 day average self-monitored blood glucose (mg/dL)		106 (102, 113)	108 (104, 112)	0.263
Unknown		4	0	
90 day average self-monitored blood glucose SD		18.7 (16.1, 22.3)	19.2 (17.2, 22.3)	0.475
Unknown		4	2	
Dietitian management		27 (29%)	29 (31%)	0.786
Urinary ketones		16 (18%)	14 (16%)	0.714
Gestational age at anatomical scan (weeks)		20.00 (20.00, 20.00)	20.00 (20.00, 20.00)	0.728
Unknown		15	14	
AC_anatomical scan (mm)		160 (153, 165)	160 (155, 164)	0.950
Unknown		15	14	
AC_percentile (Hadlock) (%)		77 (47, 90)	76 (49, 93)	0.877
Unknown		16	14	
Gestational age at last scan in third trimester (weeks)		36.00 (35.00, 37.00)	36.00 (35.00, 37.00)	0.333
Unknown		15	11	
EFW (grams)		2915 (2651, 3223)	3014 (2681, 3252)	0.461
Unknown		21	16	
EFW percentile (Hadlock) (%)		73 (47, 85)	64 (38, 82)	0.398
Unknown		35	31	
AC_last scan (mm)		326 (311, 340)	324 (311, 341)	0.725
Unknown		30	26	
AC_last scan percentile (Hadlock) (%)		80 (54, 93)	70 (52, 90)	0.385
Unknown		31	27	
Number of diabetologic consultations (n)		5.00 (4.00, 6.00)	6.00 (5.00, 7.00)	0.000
Number of fetal growth scans in pregnancy (n)		5.00 (4.00, 6.00)	3.00 (2.00, 4.00)	0.000
At least one consultation without considering fetal growth pattern (n)		29 (31%)	93 (100%)	0.000
DELTA_AC_HADLOCK		2 (−5, 11)	2 (−14, 22)	0.963
Unknown		36	38	
IOL interruption	9 (28%)		8 (26%)	0.836
IOL duration (min)	1685 (707, 2967)		1498 (703, 2407)	0.143
Unknown	6		7	
Active first stage of labor duration (min)	125 (71, 235)		125 (54, 180)	0.535
Unknown	16		15	
Second stage of labor duration (min)	22 (11, 51)		28 (11, 61)	0.735
Unknown	19		18	
Induction – labor onset duration (min)	1260 (460, 2311)		996 (442, 2013)	0.362
Unknown	12		13	
Spontaneous vaginal delivery	71 (76%)		73 (78%)	0.726
Operative vaginal delivery	4 (4.3%)		3 (3.2%)	1.000
Cesarean delivery	18 (19%)		17 (18%)	0.851
Robson				0.711
2 (Nulliparous women with a single cephalic pregnancy, ≥ 37 weeks, induced labor or pre-labor cesarean section)	40 (43%)		37 (40%)	
4 (Multiparous women without previous cesarean, single cephalic pregnancy, ≥ 37 weeks, induced labor or pre-labor cesarean section)	46 (49%)		51 (55%)	
5 (All multiparous women with at least one previous cesarean section, single cephalic pregnancy, ≥ 37 weeks)	7 (7.5%)		5 (5.4%)	
Apgar 1 min	8.00 (8.00, 9.00)		8.00 (8.00, 9.00)	0.687
Apgar 5 min				0.660
4	1 (1.1%)		0 (0%)	
6	1 (1.1%)		0 (0%)	
7	1 (1.1%)		2 (2.2%)	
8	8 (8.7%)		11 (12%)	
9	59 (64%)		53 (58%)	
10	21 (23%)		26 (28%)	
Umbilical artery pH at birth	7.28 (7.24, 7.32)		7.28 (7.24, 7.32)	0.788
Unknown	11		9	
Umbilical artery BE at birth	−2.75 (−5.10, −1.30)		−3.60 (−5.30, −1.80)	0.342
Unknown	43		47	
Neonatal weight at birth (grams)	3495 (3180, 3760)		3525 (3295, 3745)	0.367
Neonatal weight percentile (Bertino) (%)	60 (28, 86)		64 (43, 86)	0.192
LGA neonates	20 (22%)		14 (15%)	0.255
SGA neonates	5 (5.4%)		3 (3.2%)	0.721
NICU admission	2 (3.5%)		1 (1.7%)	0.615
Post-partum hemorrhage	18 (20%)		17 (18%)	0.823

^*1*^ Median (Q1, Q3); n (%)

^*2*^ Wilcoxon rank sum test; Fisher’s exact test; Wilcoxon rank sum exact test; Pearson’s Chi-squared test

ART = assisted reproductive technology; GDM = gestational diabetes mellitus; OGTT = Oral glucose tolerance test; SD = standard deviation; AC = abdominal circumference; EFW = estimated fetal weight; IOL = induction of labor; LGA = Large for gestational age; SGA = small for gestational age; NICU = neonatal intensive care unit

In the sensitivity analysis restricted to insulin-treated patients, the results remained consistent with those of the primary analysis (Table 3).

**Table 3:** Sensitivity analysis restricted to insulin-treated patients (diet-treated patients excluded) in the propensity score–matched cohort

Characteristic	Fetal growth guided -management N = 65^*1*^	Standard management N = 76^*1*^	p-value^*2*^
ART pregnancy	4 (15%)	4 (11%)	0.715
Maternal age at delivery (years)	33.0 (30.0, 37.0)	35.0 (30.0, 38.0)	0.139
Gestational age at delivery (weeks)	39.00 (39.00, 39.00)	39.00 (39.00, 39.00)	0.512
Gestational age at delivery time (total days)	276.0 (274.0, 279.0)	276.0 (273.0, 278.0)	0.258
Pre-gestational weight (kg)	74 (64, 85)	75 (63, 90)	0.882
Unknown	19	20	
Maternal weight at first diabetologic consulation (kg)	83 (72, 94)	80 (70, 92)	0.535
Maternal weight at delivery time (kg)	84 (75, 96)	84 (75, 98)	0.820
Unknown	0	1	
Delta weight (kg)	11.9 (7.8, 14.6)	11.1 (6.5, 13.4)	0.473
Unknown	34	40	
Previous_GDM	17 (26%)	25 (33%)	0.355
Unknown	0	1	
OGTT_T0 (mg/dL)	95.0 (93.0, 100.0)	96.0 (93.0, 100.5)	0.721
OGTT_T60 (mg/dL)	157 (136, 183)	157 (140, 184)	0.773
OGTT_T120 (mg/dL)	126 (110, 147)	125 (110, 145)	0.965
HbA1c (%)	5.25 (4.90, 5.50)	5.30 (4.90, 5.50)	0.860
Unknown	43	42	
Basal Insulin (IU)	60 (92%)	74 (97%)	0.248
Prandial Insulin (IU)	16 (25%)	14 (18%)	0.370
Total Insulin dosage at delivery (IU)	12 (8, 24)	17 (8, 26)	0.303
90 day average self-monitored blood glucose (mg/dL)	109 (105, 115)	109 (105, 113)	1.000
90 day average self-monitored blood glucose SD	18.6 (16.4, 22.6)	19.5 (17.3, 22.7)	0.379
Dietitian management	24 (37%)	21 (28%)	0.238
Urinary ketones	13 (20%)	12 (16%)	0.533
Gestational age at anatomical scan (weeks)	20.00 (20.00, 20.00)	20.00 (20.00, 20.00)	0.290
Unknown	10	12	
AC_anatomical scan (mm)	160 (153, 166)	160 (154, 164)	0.681
Unknown	10	12	
AC_percentile (Hadlock) (%)	75 (44, 92)	77 (44, 92)	0.863
Unknown	11	12	
Gestational age at last scan in third trimester (weeks)			0.489
31	1 (1.8%)	1 (1.5%)	
32	1 (1.8%)	2 (2.9%)	
33	4 (7.1%)	1 (1.5%)	
34	6 (11%)	8 (12%)	
35	6 (11%)	14 (21%)	
36	20 (36%)	22 (32%)	
37	11 (20%)	16 (24%)	
38	5 (8.9%)	4 (5.9%)	
39	2 (3.6%)	0 (0%)	
Unknown	9	8	
EFW (grams)	2888 (2697, 3117)	3019 (2722, 3283)	0.182
Unknown	13	12	
EFW percentile (Hadlock) (%)	73 (47, 84)	65 (40, 82)	0.555
Unknown	23	25	
AC_last scan (mm)	327 (311, 340)	324 (314, 342)	0.837
Unknown	18	20	
AC last scan percentile (Hadlock) (%)	82 (57, 93)	71 (56, 91)	0.519
Unknown	19	21	
Number of diabetologic consultations (n)	5.00 (4.00, 6.00)	6.00 (5.00, 7.00)	0.008
Number of fetal growth scans in pregnancy (n)	5.00 (4.00, 6.00)	3.00 (2.50, 5.00)	0.000
At least one consultation without considering fetal growth pattern (n)	23 (35%)	76 (100%)	0.000
DELTA_AC_HADLOCK	3 (−4, 11)	3 (−14, 22)	0.997
Unknown	24	30	

IOL interruption (n)	4 (18%)	4 (17%)	1.000
IOL duration (min)	1802 (668, 2984)	1529 (735, 2407)	0.283
Unknown	5	6	
Active first stage of labor duration (min)	121 (60, 226)	123 (56, 192)	0.848
Unknown	9	12	
Second stage of labor duration (min)	21 (10, 48)	20 (10, 67)	0.564
Unknown	12	13	
Induction – labor onset duration (min)	1395 (445, 2660)	1040 (590, 2060)	0.565
Unknown	6	10	
Spontaneous vaginal delivery (n)	52 (80%)	60 (79%)	0.877
Operative vaginal delivery (n)	2 (3.1%)	3 (3.9%)	1.000
Cesarean delivery (n)	11 (17%)	13 (17%)	0.977
Robson			0.732
2 (Nulliparous women with a single cephalic pregnancy, ≥ 37 weeks, induced labor or pre-labor cesarean section)	26 (40%)	28 (37%)	
4 (Multiparous women without previous cesarean, single cephalic pregnancy, ≥ 37 weeks, induced labor or pre-labor cesarean section)	33 (51%)	43 (57%)	
5 (All multiparous women with at least one previous cesarean section, single cephalic pregnancy, ≥ 37 weeks)	6 (9.2%)	5 (6.6%)	
Apgar 1 min	8.00 (8.00, 9.00)	8.00 (8.00, 9.00)	0.653
Apgar 5 min			0.276
4	1 (1.6%)	0 (0%)	
6	1 (1.6%)	0 (0%)	
7	1 (1.6%)	1 (1.3%)	
8	4 (6.3%)	10 (13%)	
9	43 (67%)	43 (57%)	
10	13 (20%)	21 (28%)	
Umbilical artery pH at birth	7.28 (7.23, 7.31)	7.29 (7.24, 7.32)	0.513
Unknown	9	7	
Umbilical artery BE at birth	−2.90 (−5.50, −1.50)	−3.60 (−5.30, −1.95)	0.825
Unknown	26	36	
Neonatal weight at birth (grams)	3460 (3132, 3725)	3522 (3275, 3738)	0.372
Neonatal weight percentile (Bertino) (%)	59 (29, 84)	64 (43, 87)	0.196
LGA neonates	11 (17%)	11 (14%)	0.690
SGA neonates	3 (4.6%)	3 (3.9%)	1.000
NICU admission	1 (2.5%)	1 (2.1%)	1.000
Post-partum hemorrhage	16 (25%)	13 (17%)	0.251

1 Median (Q1, Q3); n (%)

2 Wilcoxon rank sum test; Fisher’s exact test; NA; Pearson’s Chi-squared test; Wilcoxon rank sum exact test

*ART* assisted reproductive technology, *GDM* gestational diabetes mellitus, *OGTT* Oral glucose tolerance test, *SD* standard deviation, *AC* abdominal circumference, *EFW* estimated fetal weight, *IOL* induction of labor, *LGA* Large for gestational age, *SGA* small for gestational age, *NICU* neonatal intensive care unit

## Discussion

### Main findings

In this study, after propensity score matching a fetal growth-guided approach to GDM management was not associated with significant differences in obstetric and neonatal outcomes compared with standard management, including mode of delivery, labor progression and neonatal condition at birth. However, fetal growth-guided management was associated with lower use of basal insulin and differences in care processes, reflecting a more tailored therapeutic approach. The differences observed in the overall cohort, such as higher maternal weight and slightly greater neonatal birthweight in the standard management group, were attenuated after matching, suggesting that these findings were primarily driven by baseline imbalances rather than the management strategy itself. These results were confirmed in a sensitivity analysis restricted to patients requiring insulin therapy, which similarly showed no significant differences in maternal or neonatal outcomes between groups. Overall, these findings suggest that integrating fetal growth assessment into GDM management may support a more personalized approach without compromising maternal and neonatal outcomes.

## Strengths and limitations

A key strength of this study is the use of a personalized, fetal growth-guided approach to GDM management, which accounts for the substantial variability in fetal response to the diabetic intrauterine environment. Another important strength is the multidisciplinary care model, in which diabetologists are responsible for pharmacological management while obstetricians assess fetal growth, allowing for an integrated and clinically coherent decision-making process. By applying propensity score matching based on a multivariable model, we were able to isolate the specific impact of growth-guided management on insulin requirements. The comprehensive assessment of maternal and neonatal outcomes further supports the safety and clinical relevance of this strategy. Another strength of this study is the inclusion of a sensitivity analysis restricted to insulin-treated patients, which confirmed the consistency of the findings and supports the robustness of the results by reducing potential heterogeneity related to the inclusion of cases managed with diet only.

However, several limitations should be acknowledged. First, the retrospective, single-center design limits the external validity. The study population was derived from an institutional database dedicated to IOL, and the analysis was therefore restricted to women with GDM undergoing IOL. While this allowed the evaluation of a relatively homogeneous population in terms of delivery management and timing, it may limit the generalizability of the findings to the broader GDM population. Second, exposure was defined based on the documentation of fetal growth parameters across diabetological consultations rather than on a prospectively defined management protocol. Although, in our clinical setting, the availability of fetal biometry generally reflects the incorporation of ultrasound findings into clinical decision-making, this cannot be formally verified. As a result, misclassification and information bias cannot be excluded. This limitation cannot be fully addressed through propensity score matching. Third, maternal weight at the first diabetologic consultation was used in the propensity score model as a proxy for anthropometric status, due to incomplete height data precluding body mass index (BMI) calculation. Finally, despite the absence of significant differences in SGA rates after matching, the study may be insufficiently powered to identify modest but clinically relevant differences in infrequent outcomes. Therefore, a residual risk of SGA cannot be excluded. Overall, these limitations warrant cautious interpretation of the findings and highlight the need for prospective studies to better assess the causal impact of fetal growth-guided management in GDM.

## Interpretation

The integration of fetal ultrasound parameters into GDM management has long been explored as a strategy to tailor treatment intensity. Early randomized studies demonstrated that incorporating fetal AC into decision-making could reduce unnecessary insulin therapy without worsening neonatal outcomes. For example, Kjos et al. randomized women with GDM and fasting hyperglycemia (FPG, 105–120 mg/dl) to either a standard approach or an experimental strategy guided by maternal glycemia and fetal AC. In the experimental group, insulin was initiated when fetal AC reached or exceeded the 70th percentile and/or when FPG exceeded 120 mg/dl [13], allowing avoidance of insulin in a substantial proportion of women without increasing neonatal morbidity. Similarly, subsequent randomized studies adjusting glycemic targets according to fetal biometry showed that treatment intensity could be modulated based on fetal growth. Bonomo et al. reported improved perinatal outcomes, including reductions in LGA, SGA, and macrosomia, with an ultrasound-guided approach based on fetal AC percentiles [14]. Another study comparing standard management with an ultrasound-based strategy, in which insulin was initiated if fetal AC exceeded the 75th percentile, found similar maternal and neonatal outcomes [15]. More recently, an RCT showed that adjusting GDM treatment based on fetal growth reduced insulin use without worsening glycemic control or neonatal outcomes [16]. A meta-analysis by Fernández-Alonso et al. confirmed that ultrasound-guided management can safely optimize pharmacologic intervention while maintaining comparable outcomes [17]. Our findings are consistent with this evidence and reinforce that glycemia-based management alone may not capture the heterogeneity of fetal response, potentially leading to overtreatment. An important aspect to consider when interpreting our findings is the heterogeneity of approaches adopted in previous studies. In particular, substantial variability exists in both the AC thresholds used to guide treatment intensification and the glycemic targets applied according to fetal growth. Several studies have employed higher AC cut-offs and more permissive glycemic targets in pregnancies with normal fetal growth, thereby potentially reducing treatment intensity more markedly. In our study, glycemic targets were also adapted according to fetal growth, with different therapeutic thresholds applied based on whether AC was above or below the 70th percentile. This approach is consistent with a personalized management strategy but may differ from other studies in the specific thresholds adopted and in the degree of target relaxation. It is plausible that the use of more permissive glycemic targets in fetuses with normal growth contributes to lower insulin requirements. Nevertheless, our findings demonstrate that a fetal growth-guided approach, including the modulation of glycemic targets according to AC, is associated with reduced insulin use without adversely affecting maternal or neonatal outcomes. These results support the feasibility of integrating fetal biometry into GDM management within a personalized therapeutic framework.

Last, but not least important, the sensitivity analysis restricted to insulin-treated patients yielded findings consistent with the primary analysis, confirming the absence of significant differences in maternal and neonatal outcomes between the two groups. However, the fetal growth-guided approach was associated with a higher intensity of monitoring, including a greater number of ultrasound assessments and clinical consultations. This raises the question of whether such a strategy achieves an optimal balance between resource utilization and clinical benefit. At the same time, it should be recognized that the study may be underpowered to detect small but clinically meaningful differences in obstetric and neonatal outcomes, particularly for relatively infrequent events. Therefore, the absence of statistically significant differences should be interpreted with caution and does not necessarily imply equivalence between the two management strategies.

## Conclusion

In conclusion, our findings suggest that a personalized management approach to GDM based on fetal growth assessment is associated with reduced insulin requirements and a slightly longer gestational duration, without increasing operative delivery rates or adverse neonatal outcomes. By incorporating fetal biometry as an additional clinical filter, this strategy enables a more refined interpretation of maternal metabolic status and supports more appropriate therapeutic decisions. However, randomized clinical trials evaluating the efficacy of glycemic cut-offs adjusted for fetal AC percentiles or the presence of polyhydramnios, and their influence on maternal and fetal outcomes, are warranted to inform evidence-based practice.

## Data Availability

The data supporting the findings of this study are not publicly available due to privacy and confidentiality concerns but can be made available in anonymized form from the corresponding author upon reasonable request and subject to approval by the relevant ethics committee.
